# Growth Differentiation Factor-15 (GDF-15) Is Associated With Mortality in Ischemic Stroke Patients Treated With Acute Revascularization Therapy

**DOI:** 10.3389/fneur.2019.00611

**Published:** 2019-06-14

**Authors:** Céline Brenière, Alexandre Méloux, Martin Pédard, Christine Marie, Pierre Thouant, Catherine Vergely, Yannick Béjot

**Affiliations:** ^1^Equipe d'Accueil (EA 7460): Physiopathologie et Epidémiologie Cérébro-Cardiovasculaires (PEC2), University Bourgogne Franche-Comté, UFR Sciences de Santé, Dijon, France; ^2^Department of Neurology, University Hospital of Dijon, Dijon, France; ^3^INSERM UMR1093-CAPS, University Bourgogne Franche-Comté, UFR Sciences de Santé, Dijon, France; ^4^Department of Neuroradiology, University Hospital of Dijon, Dijon, France

**Keywords:** stroke, GDF15, mortality, thrombectomy, thrombolysis

## Abstract

**Background and Aims:** Growth differentiation factor-15 (GDF-15) has been identified as a robust marker of developing cardiovascular disease, however, little is currently known about its prognostic value in stroke patients. In a context of growing interest to discover new biomarkers in stroke, we aimed to assess the association between circulating GDF-15 levels and three-month mortality in ischemic stroke patients treated with acute revascularization therapy.

**Methods:** 173 patients hospitalized for acute ischemic stroke and treated with either intravenous thrombolysis (*n* = 99, 57.2%), mechanical thrombectomy (*n* = 41, 23.4%) or combined therapy (*n* = 33, 19.1%) were prospectively included. Baseline clinical and biological characteristics were recorded. Plasma GDF-15 levels were measured at admission (D0), and at 24 h, 3 and 7 days. Clinical severity was assessed with the National Institutes of Health Stroke Scale (NIHSS) score, and vital status was obtained 3 months after the stroke.

**Results:** At 3 months post-stroke, 32 patients (18.5%) had died. The deceased patients had higher D0 plasma GDF-15 levels (median [IQR]: 2,777 [1,769–5,446] vs. 1,460 [965–2,079] pg/mL, *P* < 0.001). In multivariable logistic regression analysis, D0 GDF-15 levels in the third tertile of the distribution were independently associated with mortality at 3 months (OR = 3.71; 95% CI: 1.09–12.6, *P* = 0.036), even after adjustment for confounding variables including clinical severity.

**Conclusions:** Our data show for the first time that GDF-15 plasma concentration at admission is independently associated with 3-month mortality in ischemic stroke patients treated with acute revascularization therapy. The pathophysiological mechanisms that could explain this association warrant further study.

## Introduction

Extensive research has contributed to the identification and routine use of several blood biomarkers in the field of cardiovascular and metabolic diseases, but the recognition of such biomarkers for ischemic stroke patients remains challenging. One potential biomarker is growth differentiation factor 15 (GDF-15), a cytokine belonging to the TGF-β superfamily (1). GDF-15 is weakly expressed in several tissues under physiological conditions except in the placenta (2, 3). Blood GDF-15 concentrations tend to increase with age and they do not vary with sex in apparently healthy elderly individuals, free of major medical comorbidities (4, 5). Moreover, GDF-15 plasma concentrations do increase in pathological situations and are associated with hypoxia, inflammation, oxidative stress, and oncogene activation. Elevated GDF-15 levels have also been closely associated with all-cause mortality (6–8). As a biomarker, GDF-15 has widely been studied in cardiovascular diseases (9) where it is considered as an independent marker of poor prognosis in patients with coronary artery disease (10–12), acute myocardial infarction (13–16), atrial fibrillation (17), and heart failure with preserved or reduced ejection fraction (18, 19). The cellular effects of GDF-15 may also vary according to context. For instance, GDF-15 expression increases in macrophages and may have proinflammatory effects in atherosclerosis (20, 21), whereas anti-inflammatory effects have been observed in acute diseases such as myocardial infarction (22). Moreover, GDF-15 is massively upregulated by nitrosative stress in cardiomyocytes subjected to simulated ischemia-reperfusion (23), and has been experimentally identified as a cardioprotective cytokine (22, 24). In contrast, there is little available data concerning GDF-15 involvement in ischemic stroke. In the brains of rats and mice, where GDF-15 is physiologically expressed in the choroid plexus epithelium, cortical lesioning or cerebral ischemia has been shown to induce a significant increase of GDF-15 expression in the injured cortex (25, 26). Recent data from our research unit also revealed that ischemic stroke in rats leads to a rapid and persistent increase of GDF-15 in blood circulation (27). In a more clinical context, GDF-15 could be used as a biomarker to predict stroke risk in hypertensive patients (28) as well as mortality and stroke risk in atrial fibrillation (29). Circulating GDF-15 is also associated with subclinical brain injury (30). It has been proposed that GDF-15 could be associated with poor functional outcomes after ischemic stroke (31–33). However, whether GDF-15 could be a biomarker of mortality after acute ischemic stroke treated with acute revascularization therapy remains to be elucidated. The main objective of our study was therefore to assess the association between circulating levels of GDF-15 and three-month all-cause mortality in ischemic stroke patients treated with thrombolysis and/or mechanical thrombectomy.

## Methods

### Study Population

The PARADISE study (Prognosis After Revascularization therapy in the Dijon Ischemic Stroke Evaluation study; Clinical trial NCT02856074) is an ongoing hospital-based cohort of consecutive patients with acute ischemic stroke treated with either intravenous thrombolysis (IVT) and/or mechanical thrombectomy, and who were admitted to the intensive care stroke unit of the Dijon University Hospital (Burgundy, France). For the present study, analyses were conducted on patients included between January 2017 and September 2017 for whom blood samples were collected. For each patient, ischemic stroke was diagnosed by a senior stroke-trained neurologist based on symptoms and a clinical evaluation, and confirmed by brain imaging (either cerebral computed tomography or magnetic resonance imaging). Acute revascularization therapy was performed in routine practice and based on the physician's application of the current recommendations. Ischemic stroke was classified using TOAST (Trial of ORG 10172 in Acute Stroke Treatment) criteria (34), according to results of the diagnostic work-up performed during hospitalization.

### Data Collected and Outcome Measured

At inclusion, demographics and vascular risk factors were recorded: hypertension (high blood pressure reported in a patient's medical history or patients taking antihypertensive treatment), diabetes mellitus (glucose level ≥ 7.8 mmol/L reported in the medical record or patients under insulin or oral hypoglycaemic agents), hypercholesterolemia (total cholesterol level ≥ 5.7 mmol/L reported in the medical history or patients treated with lipid-lowering therapy), and current smoking. History of stroke, transient ischemic attack (TIA), myocardial infarction, peripheral artery disease, heart failure, and atrial fibrillation were also recorded. Stroke severity was evaluated using the National Institutes of Health Stroke Scale (NIHSS) score at admission (Day 0, D0), at 24 h (Day 1, D1), and at discharge. C-reactive protein (CRP) and creatinine levels were obtained from routine blood samples at admission.

The primary outcome was all-cause mortality at 3 months. Information about vital status was systematically obtained from patients and/or their relatives by telephone.

### GDF-15 Measurement

Prior to the beginning of the study, we evaluated GDF-15 in blood samples stored at 4°C for a maximum duration of 24 h to establish the good stability of the biomarker in standard hospital storage conditions. We also included a standard pool of plasma in each set of measurement so as to exclude possible inter-experiment variations. Furthermore, we did not observe any effects on GDF-15 concentrations of long-term storage of samples at −80°C (9 months). For this study, 2 mL of blood were collected at admission as soon as possible after reperfusion therapies (Day 0, D0), 24 h (±2 h) after the first sample (Day 1, D1), at 3 days (Day 3, D3), and at 7 days (Day 7, D7). All blood samples were then quickly centrifuged at 3,500 rpm at 4°C for 5 min. Plasma samples were stored in aliquots at −80°C for a maximum of 3 months until assayed. GDF-15 levels were measured with an Enzyme-linked Immuno-Sorbent Assay (ELISA) Development kit from R&D Systems (Lille, France) according to manufacturer protocol. The detection limit was 23.4 pg/mL. The operators performing the measurements were blinded to treatment and patient outcomes.

### Statistical Analyses

Baseline characteristics were given as frequencies for categorical variables, and as means, standard deviation, medians and interquartile ranges for continuous variables. Proportions and mean values were compared (for patients alive vs. dead at 3 months) using the Chi-Square Test, the Fisher Exact test, and the Wilcoxon-Mann-Whitney test when appropriate. We used the Wilcoxon signed-rank test to compare the circulating levels of GDF-15 at the various points in time and the Spearman rank correlation analysis to assess the correlations between GDF-15 levels and clinical severity (NIHSS score), age, and all vascular risk factors and previous vascular diseases. A Bonferroni correction was used to account for multiple comparisons. Logistic regression analysis was used to evaluate the association between GDF-15 and mortality at 3 months. GDF-15 levels were analyzed according to the tertiles of their distribution (third tertile vs. first and second tertiles) because they did not meet the normality assumption. In multivariable models, we introduced age, sex, and all baseline characteristics for which a *P*-value < 0.20. Additionally, receiver operator characteristic (ROC) analysis was performed, and the c-statistic representing the area under the ROC curve for the models was evaluated. We compared discrimination of models with and without GDF-15 variables by calculating the integrated discrimination improvement (IDI). *P*-Values < 0.05 were considered statistically significant. Statistical analyses were performed with STATA@13 software (StataCorp LP, College Station, Texas, USA).

### Ethics

The PARADISE study was approved by the Institutional Review Board (CPP EST I: 2015/32; IDRCB: 2015-A01664-45). Oral informed consent was collected from the patients or their relatives according to French Legislation.

## Results

### Baseline Characteristics of Study Population

Over the study period, 175 patients with acute ischemic stroke treated with revascularization therapy were enrolled; two were subsequently excluded because 3-month vital status could not be confirmed. The final cohort consisted of 173 patients (mean age 74.6 ± 13.6 years, 49% women): 99 (57.2%) received IVT alone, 41 (23.4%) underwent mechanical thrombectomy alone, and 33 (19.1%) received a combined therapy.

At 3 months, 32 patients (18.5%) had died. The baseline characteristics of patients grouped by vital status at 3 months are shown in Table 1. Patients who died were older, and were more likely to have hypertension, history of stroke, peripheral artery disease, and heart failure. They had a greater NIHSS score at admission and at 24 h, and higher levels of CRP at admission than surviving patients. In addition, data about premorbid Rankin score (mRS) was available for 162/173 (93.6%) patients, including 138/141 (97.8%) patients alive at 3 months, and 24/32 (75%) patients dead at 3 months. Among overall patients, 25.9% had a prior-to-stroke mRS score ≥ 2 (21% in patients alive at 3 months vs. 54.2% in dead patients, *p* = 0.001).

**Table 1:** Characteristics of patients according to vital status at 3 months.

	**All patients (*n* = 173)**	**Alive (*n* = 141)**	**Dead (*n* = 32)**	***P***
**Age (years)**				
Median (IQR)	76.9 (66.3–85.1)	75.9 (65.7–82.9)	85.4 (73.1–88.5)	
First tertile (*n* = 58)		52 (36.9%)	6 (18.8%)	0.050
Second tertile (*n* = 58)		51 (36.2%)	7 (21.9%)	0.122
Third tertile (*n* = 57)		38 (26.9%)	19 (59.4%)	<0.001
Women	85 (49%)	73 (52%)	12 (38%)	0.145
NIHSS at admission	11.6 ± 6.5	10.3 ± 6.0	17.1 ± 5.8	<0.001
NIHSS at D1 (*n* = 171)	7.5 ± 6.8	5.6 ± 5.2	16.8 ± 7.1	<0.001
**Etiology (TOAST classification)**				0.039
Large artery	26 (15%)	17 (12%)	9 (28.1%)	
Cardioembolic	66 (38.2%)	50 (35.5%)	16 (50%)	
Small vessel disease	3 (1.7%)	3 (2.1 %)	0	
Other causes	6 (3.5%)	6 (4.3%)	0	
Unknown	71 (41%)	64 (45.4 %)	7 (21.9%)	
Multiple causes	1 (0.9%)	1 (0.7%)	0	
**Cardiovascular risk factors**				
Hypertension	108 (62.4%)	84 (59.6%)	24 (75%)	0.020
Diabetes mellitus	27 (15.6%)	20 (14.2%)	7 (21.9%)	0.279
Current smoking	21 (12.1%)	20 (14.2%)	1 (3.1%)	0.084
Hypercholesterolemia	58 (33.5%)	48 (34%)	10 (31.3%)	0.763
**Previous vascular diseases**				
Previous stroke	20 (11.6%)	13 (9.2%)	7 (21.88%)	0.037
Previous TIA	6 (3.5%)	6 (4.3%)	0	0.250
Myocardial infarction	13 (7.5%)	9 (6.4%)	4 (12.5%)	0.236
Peripheral artery disease	7 (4.1%)	3 (2.1%)	4 (12.5%)	0.007
Heart failure	17 (9.8%)	10 (7.1%)	7 (21.9%)	0.011
Atrial fibrillation	29 (16.8%)	21 (14.9%)	8 (32%)	0.167
**Stroke localization**				0.29
MCA/AChoA territory	150 (87.7%)	121 (87.0%)	29 (90.6%)	
ACA territory	4 (2.3%)	2 (1.4%)	2 (6.2%)	
PCA territory	9 (5.3%)	0 (6.5%)	0	
Cerebellum	1 (0.6%)	1 (0.7%)	0	
Brainstem	7 (4.1%)	6 (4.3%)	1 (3.1%)	
**Acute revascularization therapy**				0.151
IV Thrombolysis only	99 (57.2%)	85 (60.3%)	14 (43.8%)	
Thrombectomy only	41 (23.4%)	31 (22%)	10 (31.3%)	
Combined therapy	33 (19.1%)	25 (17.7%)	8 (25%)	
**Biological data**				
GDF-15 D0 (pg/mL)				
mean, SD	2,101 ± 1,644	1,756 ± 1,203	3,622 ± 2,353	<0.001
Median (IQR)	1,629 (1,030–2,457)	1,460 (965–2,079)	2,777 (1,769–5,446)	
CRP (mg/L)				
Median (IQR)	13.5 ± 23.9	10.1 ± 17.9	28.2 ± 38	0.001
Creatinine (μmol/L)				
Median (IQR)	72 (61–89	74 (62–86)	70 (58–93)	0.812

*MCA, middle cerebral artery; AChoA, anterior choroidal artery; ACA, anterior cerebral artery; PCA, posterior cerebral artery*.

### GDF-15 Levels According to Clinical Characteristics and Time Trends

**Figure 1:**
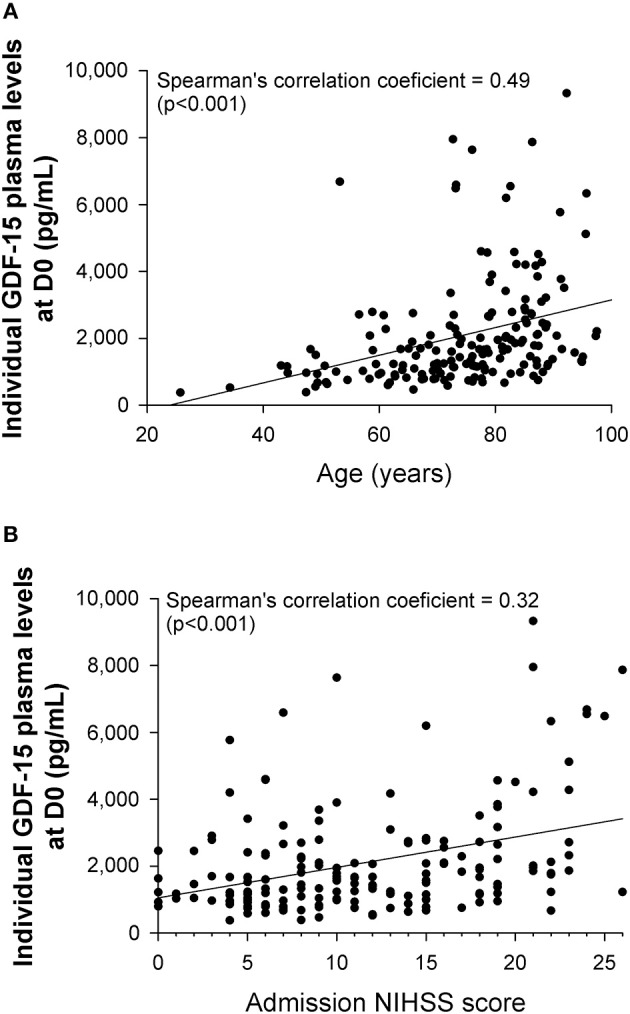
Correlation between individual GDF-15 plasma levels at admission (D0) and either **(A)** age or **(B)** admission NIHSS score (*n* = 173).

**Figure 2:**
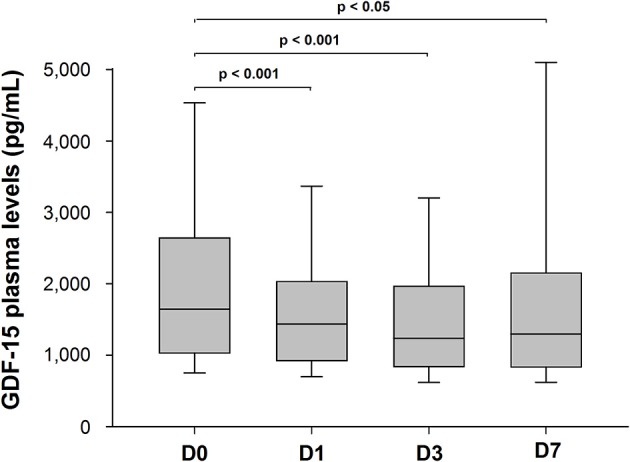
Time trends in median GDF-15 plasma levels of stroke patients at D0 (*n* = 173), D1 (*n* = 163), D3 (*n* = 124), and D7 (*n* = 45) after admission.

GDF-15 levels at admission (D0) were correlated with age (Spearman's correlation coefficient = 0.49; *P* < 0.001), initial NIHSS score (Spearman's correlation coefficient = 0.32; *P* = 0.003) (Figure 1), hypertension (Spearman's correlation coefficient = 0.38; *P* < 0.001), and diabetes (Spearman's correlation coefficient = 0.33; *P* < 0.001). In addition, GDF-15 levels at D1 were also correlated with NIHSS score at 24 h (Spearman's correlation coefficient = 0.37; *P* < 0.001; *n* = 165). GDF-15 circulating levels changed over time as follows: median [IQR] GDF-15 levels declined between D0 (1,629 [1,030–2,457] pg/mL) and D1 (1,416 [921–1,957] pg/mL) (*P* < 0.001; *n* = 163); between D0 and D3 (1,232 [841–1,951] pg/mL) (*P* < 0.001; *n* = 124), and between D0 and D7 (1,285 [836–1,892] pg/mL) (*P* = 0.019; *n* = 45) (Figure 2).

### Association Between GDF-15 Levels and Mortality at 3 Months

**Figure 3:**
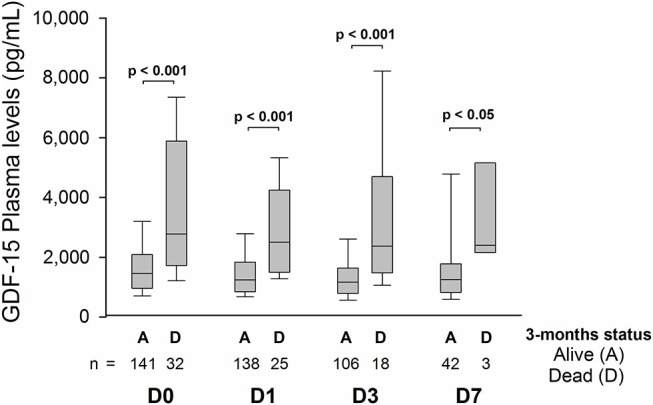
Median GDF-15 plasma levels at D0 (*n* = 173), D1 (*n* = 163), D3 (*n* = 124), and D7 (*n* = 45) according to vital status at 3 months.

**Figure 4:**
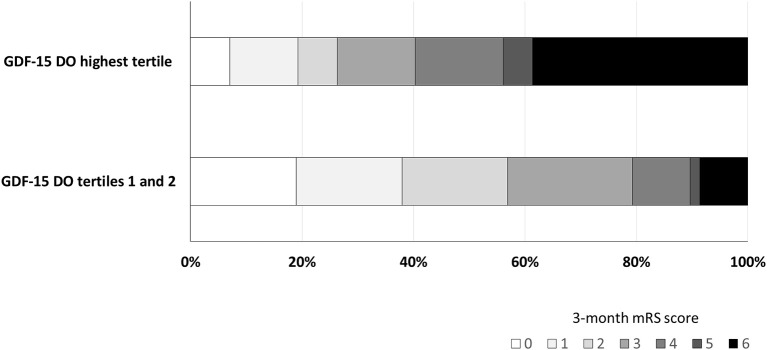
Distribution of mRS scores at 3 months according to D0 GDF-15 tertiles.

In patients who had died at 3 months, median plasma GDF-15 levels were greater at D0 (*P* < 0.001), D1 (*P* < 0.001), D3 (*P* < 0.001), and D7 (*P* = 0.041) (Figure 3). Distribution of mRS scores at 3 months (Figure 4) shows that patients in the third (highest) tertile of D0 GDF-15 were more likely to be dead (38.8 vs. 8.6%, *p* < 0.001). In univariate logistic regression analysis, GDF-15 levels in the third tertile of the distribution at all times points were associated with a higher risk of death (Table 2). In multivariable logistic regression analysis, D0 GDF-15 levels in the third tertile of the distribution were associated with mortality at 3 months (OR = 3.71; 95% CI: 1.09–12.6, *p* = 0.036) (Table 3). Addition of the premorbid mRS into the models did not alter the results. Hence, D0 GDF-15 levels in the third tertile of the distribution were associated with mortality at 3 months (OR = 4.46; 95% CI: 1.15–17.4, *p* = 0.031). No significant association between either D1 GDF-15 levels (OR = 0.33; 95% CI: 0.07–1.62, *p* = 0.17) or D3 GDF-15 levels (OR = 0.18; 95% CI: 0.01–3.71, *p* = 0.27) and mortality were noted.

**Table 2:** Association between 3-month mortality and GDF-15 levels in the third tertile at D0 (GDF > 2,088 pg/mL), D1 (GDF > 1,784 pg/mL), and D3 (GDF > 1,575 pg/mL) in univariate logistic regression analysis.

	**OR**	**95% CI**	***P*-value**
GDF-15 D0 (*n* = 173)	6.66	2.88–15.43	<0.001
GDF-15 D1 (*n* = 163)	3.81	1.58–9.20	0.003
GDF-15 D3 (*n* = 124)	5.31	1.82–15.47	0.002

**Table 3:** Multivariable logistic regression analysis of the association between 3-month mortality and GDF-15 levels in the third tertile of the distribution at D0 (GDF-15 > 2,088 pg/mL), D1 (GDF-15 >1,784 pg/mL), and D3 (GDF-15 > 1,575 pg/mL).

	**OR^§^**	**95% CI**	***P*-value**
GDF-15 D0 (*n* = 173)	3.71	1.09–12.62	0.036
GDF-15 D1 (*n* = 163)	0.62	0.16–2.34	0.478
GDF-15 D3 (*n* = 124)	0.62	0.77–4.97	0.650

§ *Adjusted for age, sex, NIHSS score at admission, hypertension, smoking, previous stroke, peripheral artery disease, heart failure, atrial fibrillation, CRP levels (third tertile), and acute revascularization therapy*.

The model without including GDF-15 showed a good discrimination (c-statistic = 0.877; 95% CI: 0.814–0.940) that was only slightly improved when adding GDF-15 (c-statistic = 0.891; 95% CI: 0.835–0.947) (IDI 0.023, *p* = 0.23).

## Discussion

This study demonstrated that, in patients hospitalized for acute ischemic stroke who received IV thrombolysis and/or mechanical thrombectomy, higher concentrations of GDF-15 at admission were associated with increased risk of death at 3 months.

We sequentially measured GDF-15 from admission to 7 days after the acute ischemic event and we observed that GDF-15 levels decreased over this period, mainly between the 24 first hours. Our results are consistent with two other studies in which GDF-15 levels declined between 6 h and 7 days after ischemic stroke in 57 patients (31) and between admission and 24 h in 264 patients (32). While we have no data concerning the pre-ischemic levels of GDF-15 in these stroke patients, we can speculate that GDF-15 concentrations were certainly elevated as a result of age and the associated cardiovascular risk factors such as hypertension and diabetes that were shown to be determinants of circulating GDF-15 levels in a large cohort study (5). As a matter of comparison, while mean GDF-15 levels in young healthy adults are around 450 pg/ml (unpublished data from preliminary experiments), the mean concentrations at admission and during hospitalization in the stroke patients presented here were approximately four times higher (2,100 ± 125 at D0 and 1,886 ± 269 at D7, mean ± SEM). Additionally, we cannot exclude the possibility that GDF-15 levels spiked during the acute cerebrovascular event as a result of ischemia and inflammation. Recent experimental data from our team revealed that ischemic stroke in rats prompted the increase in GDF-15 in the circulation 2 and 24 h after cerebral embolization (27). In pathological conditions, GDF-15 may originate from a number of tissues and in particular from inflammatory cells. However, it has been shown that cryogenic cerebral lesions induced GDF-15 expression in macrophages, neurons and microglial cells (25). Other data have also shown the induction of GDF-15 mRNA expression in the ipsilateral hippocampus and parietal cortex 3 and 24 h after cerebral ischemia induced by middle cerebral artery occlusion in mice (26). Our team has made similar unpublished observations in the ipsilateral cortex and cerebellum after cerebral embolization in rats. Therefore, we can hypothesize that the increased GDF-15 concentration in stroke patients may originate both from the pre-ischemic context of high risk factors in an elderly population and from the acute ischemic inflammatory event. Moreover, in line with a previous study (31), we found that GDF-15 levels were correlated with NIHSS score that is an appropriate rating scale for clinical severity. Our findings therefore suggest that elevated GDF-15 levels may be associated with higher clinical severity in concordance with more serious ischemic lesions.

Our study shows that GDF-15 levels at admission are associated with post-stroke mortality even after adjustment for other usual predictors, although the improvement of prediction compared with a clinical model was only limited. GDF-15 has previously been investigated as a potential biomarker of global mortality. For instance, in 1,004 elderly individuals, changes in GDF-15 concentrations between 70 and 75 years were predictors of all-cause mortality (7). In 940 71-year-old subjects followed for 10 years, an increase in GDF-15 levels was associated with a stronger risk of cardiovascular mortality, total mortality and coronary-heart-disease-related morbidity and mortality (35). GDF-15 has been recognized as a biomarker of mortality and cardiovascular events in patients with ST-elevation or non-ST-elevation acute coronary syndromes (29). In fourteen thousand five hundred and seventy-seven patients with stable coronary heart disease, the highest quartile of GDF-15 concentrations was highly associated with all-cause morbidity and with stroke (12). We observed that GDF-15 levels at admission were associated with post-stroke mortality even after adjustment for multiple variables such as NIHSS score which is a very useful scale for prognosis evaluation after stroke (36) and even after adjustment for other usual predictors. This finding is consistent with the recently published study on 3,066 ischemic stroke patients in which high GDF-15 levels were associated with unfavorable clinical outcomes including death, and major disability (33). GDF-15 may therefore have a predictive power in addition to clinical and biologic variables for the mortality risk evaluation after ischemic stroke. Our findings suggest that admission GDF-15 may be an additional biomarker to predict the outcomes of ischemic stroke patients who specifically receive acute revascularization therapy.

One of the strengths of our study is that we assessed the stability of GDF-15 levels both in blood samples stored at 4°C for a maximum duration of 24 h and in frozen plasma aliquots stored for a long-term at −80°C. Besides, in previous studies aimed at discovering different prognosis biomarkers after stroke, most of them included small groups and did not adjust for clinical information such as NIHSS or age (37). Several limitations must be acknowledged. Since samples were collected at various times after reperfusion therapies, we cannot exclude a possible source of variability due to the administration of exogenous tPA. It was not possible to clearly establish whether GDF-15 levels increase with stroke volume since no systematic MRI assessment was done. In addition, cause of death and delay of death were not recorded, thus making it impossible to specifically investigate the role of GDF-15 levels on mortality. Although the deleterious impact of elevated GDF-15 on bleeding risk has been documented, symptomatic haemorrhagic transformation was observed in only a few number of patients (2 in surviving patients and 4 in dead patients), that cannot allow to draw any definite conclusions.

To conclude, our study is the first providing data on GDF-15 time course in the plasma of post-stroke patients undergoing revascularization, either by thrombolysis or mechanical thrombectomy. Our findings further suggest that the GDF-15 cytokine could be a prognostic biomarker of mortality in ischemic stroke patients treated with acute revascularization therapy. Further studies are needed to better understand the pathophysiological mechanisms that could explain this association.

## Disclosure

YB: honoraria for lectures or consulting fees from AstraZeneca, Daiichi-Sankyo, BMS, Pfizer, Medtronic, MSD France, Amgen, and Boehringer-Ingelheim. CB, MP, CM, AM, Luc Rochette, CV: None.

## Data Availability

The raw data supporting the conclusions of this manuscript will be made available by the authors, without undue reservation, to any qualified researcher.

## Ethics Statement

The PARADISE study was approved by the Institutional Review Board (CPP EST I: 2015/32; IDRCB: 2015-A01664-45). Oral informed consent was collected from the patients or their relatives according to French Legislation.

## Author Contributions

CB: data collection, sample analysis, article writing. AM and MP: sample analysis. PT and CM: data collection and analysis. CV and YB: study design, data analysis, article writing.

### Conflict of Interest Statement

The authors declare that the research was conducted in the absence of any commercial or financial relationships that could be construed as a potential conflict of interest.
